# Assessment of the adherence and willingness to participate in colorectal cancer screening programs among people living in prison

**DOI:** 10.1371/journal.pone.0344256

**Published:** 2026-03-11

**Authors:** Giovanna Paduano, Gabriella Di Giuseppe, Mario Postiglione, Giuseppe Nese, Sabrina Morra, Maria Pavia

**Affiliations:** 1 Department of Experimental Medicine, University of Campania “Luigi Vanvitelli”, Naples, Italy; 2 U.O.C. Tutela Salute in Carcere-ASL Caserta, Caserta, Italy; University of Toronto, CANADA

## Abstract

This study aimed to assess the adherence and willingness to participate in colorectal cancer screening programs in people living in prison (PLP). This survey was conducted from October 2023 and July 2024. Of the 995 participating, 314 were eligible for colorectal cancer screening. Overall, 25.2% PLP had undergone a fecal occult blood test in a screening program. Those who reported consumption of at least 5 daily portions of fruit and vegetables, that were minimally active and had at least one chronic disease were significantly more likely to have undergone fecal occult blood test, whereas those who had a length of detention of 2–10 years, those who reported correct protein consumption, and those who do not drink alcohol were significantly less likely to have undergone a fecal occult blood test for colorectal cancer screening purposes. The majority (86%) expressed their willingness to undergo fecal occult blood test for screening in prison. Willingness was significantly higher in those involved in working activities in prison (OR = 4.18; 95% CI = 1.18–14.84; *p* = 0.027), and who had expressed willingness to receive vaccinations in prison if offered (OR = 4.4; 95% CI = 1.47–13.22; *p* = 0.008). Those at their first experience of detention and that had expressed their willingness to participate in interventions to promote healthy lifestyle were significantly less willing to undergo fecal occult blood test for screening purposes if offered in prison. This study highlights the need to promote health literacy on the role of cancer prevention in eligible PLP and the need for the elimination of organizational barriers.

## Introduction

Colorectal cancer (CRC) is the third most common cancer type and the second most common cause of cancer death worldwide [1]. In 2022, more than 1.9 million cases were diagnosed and more than 900.000 deaths per year were reported [1]. In Italy in 2023 50.500 new diagnoses and 24.200 deaths have been estimated [2].

This burden occurs despite the availability of cost-effective screening programs with evidence of reducing CRC incidence and mortality. Indeed, it has been demonstrated that trends in CRC incidence, mortality, and stage distribution across European countries appear to be largely explained by different levels of CRC screening implementation [3]. In Italy CRC screening is actively offered free of charge to all 50–69 years old subjects, but adhesion to invitation is still low (34.1% in 2022), with lower rates in the South compared to North and Central Italy (19.5% vs. 45.3% and 27.1%, respectively) [4]. Moreover, disparities in participation in CRC screening programs have been associated to social and demographic characteristics, such as education, income, race, etc [5].

It is well-known that people living in prison (PLP) disproportionally come from socially deprived and disadvantaged backgrounds and are often characterized by low education levels, high vulnerability to several health conditions, sustained frequency of untreated diseases and mental disorders, and unhealthy lifestyles, including high rates of alcohol, tobacco and drug consumption [6–12]. Moreover, the permanence in prison has been shown to deteriorate the health status of PLP [13], with high incidence of infectious diseases, such as tuberculosis, AIDS or hepatitis [14,15], mental health and eventually drug abuse [6–9,11,12]. Moreover, several studies have also documented a higher cancer prevalence among PLP, compared with the general population [16–18].

Disparities in health in this population is documented also in Italy, where, by legislation, health services provided to PLP should be the same as those provided to the general population [19].

Therefore, from a public health perspective, incarceration offers a potentially high impact opportunity to reach an underserved population, to discover preventive and treatment needs and to provide preventive and healthcare services, thus contrasting health disparities in this underprivileged population. However, little is known about the prevention needs of this population, as well as the PLP willingness to participate to prevention activities during incarceration.

Within a large project aimed at the assessment of primary and secondary prevention needs of PLP in Italy, this study was designed with the following aims: 1) to assess adhesion to CRC screening programs in males PLP; 2) to assess whether CRC screening programs are provided in prison; 3) to assess willingness of males PLP to participate in CRC screening programs if they are organized in prison.

## Materials and methods

### Setting and participants

This cross-sectional study was part of a larger project developed and conducted by the University of Campania “Luigi Vanvitelli” and the Joint Operational Unit for Health Protection at Prison Institutions to assess primary and secondary prevention needs among Italian PLP. The study was conducted from 10-11-2023, to 30-09-2024, among eligible (50–69 years) males PLP in three prisons located in the Campania region, in the South of Italy. Each selected prison hosted both people awaiting trial and those who were definitively convicted, serving short or long verdicts, and consisted of sections that hosted different groups according to low, medium or high security regimes.

### Data collection

Before starting the survey, the directors of each prison were contacted to arrange an informative meeting, during which they received a letter outlining the study’s aims, the research protocol, and a request for consent to conduct the study. Once authorization was obtained, a stratified sampling method was used to randomly select the study population. Specifically, eligible PLP were stratified by their detention status. The strata were formed based on low, medium, or high security regime of PLP and a proportional number of subjects were randomly selected from each group. Then, each potential participant was approached by trained investigators for a face-to-face interview on different days and at various times throughout the week, ensuring the inclusion of both working and non-working PLP. No compensation was provided for participation. PLP housed in special units or unable to give informed consent due to substantial cognitive impairment were excluded from the survey. Each participant received an oral standardized explanation of the aims of the interview and was invited to give verbal consent to participation. Following the participants’ verbal consent, which was asked in the presence of a correctional officer, who acted as a witness, a trained researcher conducted a face-to-face survey with each participant in a semiprivate area in the jail, with interviewer and participant seated on opposite sides of a table. Verbal consent form was judged to be the most appropriate and effective option to ensure comprehension and maximize participation, since there is ample evidence supporting alternative approaches to written consent in detained population, in order to enhance understanding and trust [20,21]. Moreover, fear of signing documents has been identified as a barrier to participation in prison-based studies [21], and low literacy levels, language barriers, and limited comprehension skills in this population have been reported to lead to misunderstanding or mistrust if written forms are used [22]. The interview was voluntary and strictly confidential, and participants were informed that declining or discontinuing participation would not affect their access to healthcare services nor their prison conditions. Each participant had an identification code, and only an officer had the list of codes associated with the participants’ names. A correctional officer remained at a distance of approximately 10 meters during the interviews to ensure safety and privacy. The interview and the compilation of the questionnaire by the research investigators lasted approximately 10 minutes.

The study protocol and the questionnaire were approved by the Ethics Committee of the University of Campania “Luigi Vanvitelli” (protocol code: N.0029553/ October 12, 2023).

### Survey instrument

The questionnaire was prepared to be administered by interview. The items in the survey were based on previous studies conducted by the research group and on an extensive literature search focused on prison settings [13,23–29]. The questionnaire consisted of four sections, preceded by an introduction illustrating the objectives of the survey and the precautions taken so that through the information contained it would not be possible to trace those who participated. The first section investigated socio-demographic and anamnestic characteristics of PLP, including detention-related information (age, nationality, marital status, number of sons/daughters, sexual orientation, educational status, employment status before and during detention, number of incarcerations, total months spent in jail, living arrangements [individual or shared cells], the presence or history of underlying chronic conditions and weight and height for Body Mass Index (BMI) calculation); the second was on lifestyle behaviors (physical activity, smoking, alcohol intake, daily consumption of fruits and vegetables, daily portions of dietary protein sources and frequency of consumption of snacks and sweets); the third was on participation in the organized CRC screening programs through the fecal occult blood test (FOBT), with specific attention to the role played by health services dedicated to the prison population in offering the screening programs, as well as on the willingness to participate in them if they were offered in prison and on the reasons for their choices; finally, in the fourth section, willingness to uptake recommended vaccination (Hepatitis B, Diphteria/tetanus/pertussis, Meningococcus ACW135Y, Meningococcus B, Measles/mumps/rubella/varicella, Hepatitis A, Pneumococcal, Influenza and Herpes Zoster) in the eligible subjects, if offered in prison, was also investigated.

In all sections, information was collected using closed-ended questions with multiple-choice answers. Alcohol-related disorders were assessed using the AUDIT-C scale [30], a validated questionnaire for predicting hazardous alcohol consumption, in a shortened version that incorporated only the first three questions: “How often do you consume alcoholic drinks?”; “On days when you drink, how many alcoholic drinks do you consume on average?”; “How often do you drink six or more glasses of alcohol on a single occasion?”. Each answer was scored from 0 to 4, with a total score ranging from 0 to 12. A score of 5 or higher is associated with an above-average risk of developing an alcohol-related disorder in men. Physical activity levels were assessed using the International Physical Activity Questionnaire – Short Form (IPAQ-SF) [31,32], which includes seven items that evaluate total energy expenditure per week by considering the number of days and minutes spent on vigorous physical activity (8 METs), moderate physical activity (4 METs), and walking (3.3 METs for intense pace, 3 METs for moderate intensity, and 2.5 METs for a slow pace). The IPAQ total score is expressed in MET-minutes/week, representing inactivity (< 700 MET-minutes/week), sufficient activity (700–2519 MET-minutes/week), or active/very active (≥ 2520 MET-minutes/week).

### Pilot study

Before initiating the survey, a pilot study was conducted with 50 men to ensure the correct interpretation, reliability, and feasibility of the questions. No changes were made to the survey instrument, and the results were included in the final sample.

### Statistical analysis

All analyses were performed using Stata software, version 17 [33]. First, descriptive statistics were conducted to summarize the main characteristics of the sample, using frequencies and proportions for categorical variables and mean (±standard deviation) for continuous variables. Second, Chi-square, Chi-square for trend, Student’s t-test and Fisher’s exact tests were employed to identify determinants associated with the outcomes of interest. Then, two stepwise multivariate logistic regression models with backward elimination were designed for the following outcomes: FOBT uptake in a screening program (Model 1), which was dichotomously recorded as 1 if the answer was “yes” and 0 if it was “no/do not remember”, and willingness to undertake FOBT for screening purposes if offered in prison (Model 2), which was dichotomized as 1 if the answer was “yes” and 0 if it was “no/not sure”. The following independent variables were selected for both models: Institution (Prison 1 = 1; Prison 2 = 2; Prison 3 = 3), age group, years (50–55 = 1; 56–60 = 2; > 60 = 3), marital status (unmarried/widowed/separated/divorced = 0; married/cohabitant = 1), sons/daughters (no = 0; yes = 1), education level (none/primary school = 0; middle school = 1; high school/university degree = 2), occupation before detention (no = 0; yes = 1), first detention (no = 0; yes = 1), length of detention, years (≤1 = 0;2–10 = 1; ≥ 11 = 2), working activity in prison (no = 0; yes = 1), at least one chronic disease (no = 0; yes = 1), smoking status (never smoker = 0; former smoker = 1; current smoker = 2), alcohol consumption (never = 0; not being at risk of alcohol abuse = 1; being at risk of alcohol abuse = 2), physical activity (inactive = 1; minimally active = 2; active/very active = 3), at least 5 daily portions of fruit and vegetables (no = 0; yes = 1), 2 daily portions of dietary protein sources (no = 0; yes = 1), rare consumption of snacks and sweets (no = 0; yes = 1), BMI category (underweight/healthy weight = 1; overweight = 2; obese = 3).

In the second model, FOBT uptake in a screening program (no/do not remember = 0; yes = 1), willingness to receive at least one recommended vaccination in prison (no/not sure = 0; yes = 1), and willingness to participate to at least one intervention about healthy lifestyle in prison (no/not sure = 0; yes = 1) were also added. Adjusted odds ratio (OR) and 95% confidence intervals (CI) were calculated. All reported *p* values are two-tailed and a value ≤0.05 is considered statistically significant.

## Results

### Socio-demographic, detention, anamnestic and lifestyle characteristics of the participants

Of the 1190 male PLP invited to participate to the project in the selected prisons, 995 gave consent, with a response rate of 83.6%. Among these, 314 fulfilled the requirement for CRC screening (50–69 years), and all of them were interviewed and included in the analysis. Table 1 shows socio-demographic, detention, anamnestic and lifestyle characteristics of the participating PLP. The mean age was 56.6 years (SD ± 5.2), the great majority (97.5%) were Italians, only 18.9% had obtained a high school or university degree, two thirds (67.5%) were married or cohabitant, 88.8% had at least one child, and 86.3% were employed before detention. Almost all (98.4%) lived in shared cells, 28.7% were involved in some working activity, 40.5% were in their first episode of detention, and the mean time spent in prison was 9.7 years. Overall, 37.9% of the participants reported to be affected by at least one chronic disease, with the most frequent being diabetes and cardiovascular diseases (20.1%), and only 30.6% had a healthy weight, with 23.9% classified as obese, and 45.5% as overweight. Current smokers were the most represented group (74.8%) with a mean number of 19.2 cigarettes a day, 7.6% were considered at risk of alcohol abuse according to the Audit C score, and half (56.1%) were active/very active according to the IPAQ score assessing physical activity. A large majority (79.9%) reported healthy dietary habits with regard to the consumption of at least 5 daily portions of fruit and vegetables and 62.1% consumed 2 daily portions of dietary protein sources.

**Table 1:** Socio-demographic, detention, anamnestic and lifestyle characteristics of participants.

Characteristics	Total (N:314)		Fecal occult blood test (FOBT) uptake in a screening program		Willingness to uptake FOBT in prison	
			Yes (N:79;25.2%)	No (N:235;74.8%)	Yes (N:234;86%)	No (N:38;14%)
	N	%	N (%)	N (%)	N (%)	N (%)
***Socio-demographics***						
**Age group, years**						
50-55	159	50.6	34 (21.4)	125 (78.6)	119 (86.9)	18 (13.1)
56-60	72	22.9	15 (20.8)	57 (79.2)	51 (79.7)	13 (20.3)
>60	83	26.5	30 (36.1)	53 (63.9)	64 (90.1)	7 (9.9)
			χ^2^=7.24, df=2, p=0.027		χ^2^=3.22, df=2, p=0.200	
**Nationality**						
Italians	306	97.5	77 (25.2)	229 (74.8)	230 (86.5)	36 (13.5)
Foreigners	8	2.5	2 (25)	6 (75)	4 (66.7)	2 (33.3)
			Fisher’s exact test= 0.676, df=1		Fisher’s exact test= 0.198, df=1	
**Sexual orientation**						
Heterosexuals	312	99.4	79 (25.3)	233 (74.7)	233 (86)	38 (14)
Homosexuals	2	0.6	0 (-)	2 (100)	1 (100)	0 (-)
			Fisher’s exact test= 0.560, df=1		Fisher’s exact test= 0.860, df=1	
**Marital status**						
Unmarried/widowed/separated/divorced	102	32.5	25 (24.5)	77 (75.5)	74 (85.1)	13 (14.9)
Married/cohabitants	212	67.5	54 (25.5)	158 (74.5)	160 (86.5)	25 (13.5)
			χ^2^=0.03, df=1, p=0.854		χ^2^=0.101 df=1, p=0.751	
**Sons/daughters**						
No	35	11.2	10 (25.6)	25 (71.4)	23 (85.2)	4 (14.8)
Yes	279	88.8	69 (24.7)	210 (75.3)	211 (86.1)	34 (13.9)
			χ^2^=0.24, df=1, p=0.622		Fisher’s exact test= 0.540, df=1	
**Education level**						
None	3	0.9	0 (-)	3 (100)	2 (66.7)	1 (33.3)
Primary school	61	19.4	12 (19.7)	49 (80.3)	43 (81.1)	10 (18.9)
Middle school	191	60.8	51 (26.7)	140 (73.3)	145 (86.3)	23 (13.7)
High school	50	16	13 (26)	37 (74)	38 (92.7)	3 (7.3)
University degree	9	2.9	3 (33.3)	6 (66.7)	6 (85.7)	1 (14.3)
			χ^2^trend=1.52, p=0.218		χ^2^trend=2.57, p=0.109	
***Detention***						
**Institution**						
Prison 1	139	44.2	29 (20.9)	110 (79.1)	108 (88.5)	14 (11.5)
Prison 2	58	18.5	8 (13.8)	50 (86.2)	34 (65.4)	18 (34.6)
Prison 3	117	37.3	42 (35.9)	75 (64.1)	92 (93.9)	6 (6.1)
			χ^2^=12.51, df=2, p=0.002		χ^2^=24.09, df=2, p<0.001	
**Occupation before detention**						
No	43	13.7	9 (20.9)	34 (79.1)	34 (87.2)	5 (12.8)
Yes	271	86.3	70 (25.8)	201 (74.2)	200 (85.8)	33 (14.2)
			χ^2^=0.47, df=1, p=0.492		χ^2^=0.05, df=1, p=0.823	
**First detention**						
No	187	59.5	44 (23.5)	143 (76.5)	138 (86.3)	22 (13.8)
Yes	127	40.5	35 (27.6)	92 (72.4)	96 (85.7)	16 (14.3)
			χ^2^= 0.65, df=1, p=0.419		χ^2^=0.01, df=1, p=0.900	
**Length of detention, years (292)** ^**a**^						
≤1	78	24.8	26 (33.3)	52 (66.7)	62 (89.9)	7 (10.1)
2-10	117	37.3	21 (17.9)	96 (82.1)	83 (80.6)	20 (19.4)
>10	97	33.2	27 (27.8)	70 (72.1)	72 (92.3)	6 (7.7)
			χ^2^= 6.33, df=2, p=0.042		χ^2^=6.102, df=2, p=0.047	
**Working activity in prison**						
No	224	71.3	59 (26.3)	165 (73.7)	163 (84.5)	30(15.5)
Yes	90	28.7	20 (22.2)	70 (77.8)	71 (89.9)	8 (10.1)
			χ^2^=0.58, df=1, p=0.447		χ^2^=1.37, df=1, p=0.242	
**Type of cell**						
Individual	5	1.6	0 (-)	5 (100)	5 (100)	0 (-)
Shared	309	98.4	79 (25.6)	230 (74.4)	229 (85.8)	38 (14.2)
			Fisher’s exact test =0.232, df=1		Fisher’s exact test =0.468, df=1	
***Anamnestic***						
**At least one chronic disease**						
No	195	62.1	39 (20)	156 (80)	148 (84.1)	28 (15.9)
Yes	119	37.9	40 (33.6)	79 (66.4)	86 (89.6)	10 (10.4)
			χ^2^=7.28, df=1, p=0.007		χ^2^=1.55, df=1, p=0.007	
**Cardiovascular disease**						
No	251	79.9	56 (22.3)	195 (77.7)	50 (86.2)	8 (13.8)
Yes	63	20.1	23 (36.5)	40 (63.5)	46 (90.2)	5 (9.8)
			χ^2^= 5.39, df=1, p=0.020		χ^2^= 0.41, df=1, p=0.521	
**Diabetes**						
No	251	79.9	60 (23.9)	191 (76.1)	47 (82.5)	10 (17.5)
Yes	63	20.1	19 (30.2)	44 (69.8)	49 (94.2)	3 (5.8)
			χ^2^=1.05, df=1, p=0.306		Fisher’s exact test =0.053, df=1	
**Body Mass Index (BMI) category**						
Under/healthy weight	92	30.6	27 (29.4)	65 (70.6)	66 (85.7)	11 (14.3)
Overweight	137	45.5	25 (18.3)	112 (81.7)	103 (83.1)	21 (16.9)
Obese	72	23.9	24 (33.3)	48 (66.7)	54 (91.5)	5 (8.5)
			χ^2^= 6.87, df=2, p=0.032		χ^2^= 2.35, df=2, p=0.310	
***Lifestyle***						
**Smoking status**						
Never smokers	54	17.2	18 (33.3)	36 (66.7)	43 (93.5)	3 (6.5)
Current smokers	235	74.8	53 (22.3)	182 (77.7)	176 (85)	31 (15)
Former smokers	25	8	8 (32)	17 (68)	15 (78.9)	4 (21.1)
			χ^2^=3.39, df=2, p=0.184		χ^2^=3.09, df=2, p=0.213	
**Alcohol consumption (Audit-C)**						
Never	171	54.5	42 (24.6)	129 (75.4)	137 (90.7)	14 (9.3)
Not being at risk of alcohol abuse	119	37.9	30 (25.2)	89 (74.8)	84 (82.4)	18 (17.6)
Being at risk of alcohol abuse	24	7.6	7 (29.2)	17 (70.8)	13 (68.4)	6 (31.6)
			χ^2^= 0.24, df=2, p=0.888		χ^2^= 8.82, df=2, p=0.012	
**Physical activity status (IPAQ score)**						
Inactive	40	12.7	15 (37.5)	25 (62.5)	25 (89.3)	3 (10.7)
Minimally active	98	31.2	31 (34.6)	67 (68.4)	70 (81.4)	16 (18.6)
Active/very active	176	56.1	33 (18.8)	143 (81.2)	139 (88)	19 (12)
			χ^2^= 9.27, df=2, p=0.010		χ^2^= 2.28, df=2, p=0.320	
**At least 5 daily portions of fruit and vegetables**						
No	63	20.1	8 (12.7)	55 (87.3)	53 (89.8)	6 (10.2)
Yes	251	79.9	71 (28.3)	180 (71.7)	181 (85)	32 (15)
			χ^2^= 6.49, df=1, p=0.011		χ^2^= 0.91, df=1, p=0.341	
**Two daily portions of dietary protein sources**						
No	119	37.9	35 (29.4)	84 (70.6)	82 (81.2)	19 (18.8)
Yes	195	62.1	44 (22.6)	151 (77.4)	152 (88.9)	19 (11.1)
			χ^2^= 1.84, df=1, p=0.175		χ^2^= 3.13, df=1, p=0.077	
**Rare consumption of snacks and sweets**						
No	124	39.5	26 (20.9)	98 (79.1)	98 (90.7)	10 (9.3)
Yes	190	60.5	53 (27.9)	137 (72.1)	136 (82.9)	28 (17.1)
			χ^2^= 1.91, df=1, p=0.1.67		χ^2^= 3.31, df=1, p=0.069	

^a^Number of each item may not add up to total number of study population due to missing values.

### CRC screening behavior and willingness to participate to CRC screening activities

**Fig 1:**
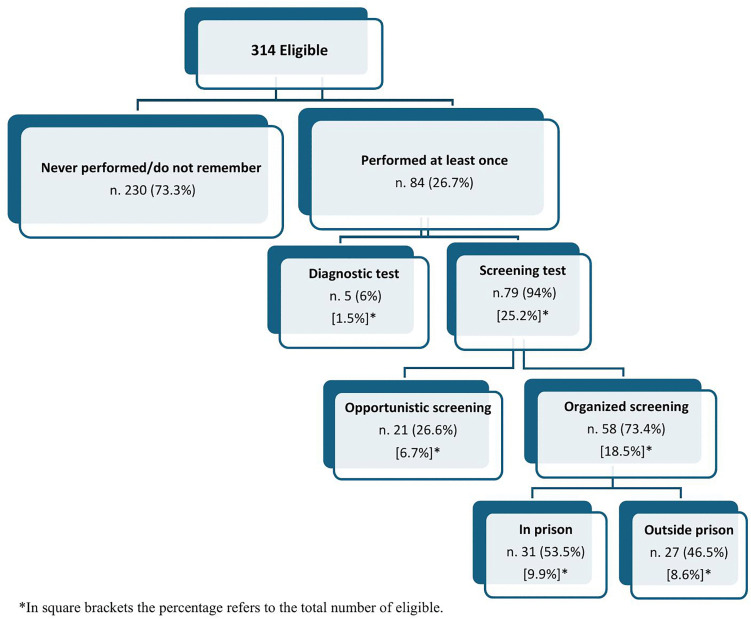
Participation in fecal occult blood test (FOBT) actiivities.

Overall, 84 (26.7%) eligible PLP had ever undergone a FOBT, 1.5% for diagnostic and 25.2% for screening purposes; for those who underwent FOBT for screening, 6.7% participated in an opportunistic procedure, and 18.5% in an organized program, 9.9% in prison and 8.6% outside the prison (Fig 1). The adherence to the screening interval of two years was reported by 43 (13.7%) of the eligible PLP. Main reported reasons for not having undergone FOBT were the perceived absence of health problems (57.1%) and not having been advised (40.6%). When PLP were asked whether they had been invited to participate in CRC screening program in prison, 40 (12.8%) reported to have been invited and all of them had undergone FOBT, whereas when those who had not been invited were asked whether they would participate in CRC screening programs if offered in prison, 86% expressed their willingness to undergo FOBT for screening purposes. Among those who were not willing to participate, the more frequent reported reasons were they were not interested to be tested (55.2%) or considered the test to be useless (20.7%), or were afraid of discovering the disease (10.3%).

Participation in CRC screening and willingness to participate in it if offered in prison according to several characteristics are reported in Table 1. Having undergone FOBT for screening purposes was significantly more likely in older PLP (p = 0.027), with at least one chronic disease (p = 0.007), who practice less physical activity (p = 0.010), are obese (p = 0.032) and consume at least 5 daily portions of fruit and vegetables (p = 0.011); moreover the participation was significantly higher for very short (< 1 year) or very long (> 10 years) duration of detention, compared to intermediate ones (2–10 years) (p = 0.045). Willingness to uptake FOBT in prison was significantly more likely in those with at least one chronic disease (p = 0.007), who have never consumed alcohol (p = 0.012), and for very long (>10 years) or very short (<1 year) duration of detention, compared to intermediate ones (2–10 years) (p = 0.047). Moreover, significant differences in previous participation (p = 0.002) and in willingness to participate (p < 0.001) in CRC screening were revealed across the different investigated prison.

### Multivariate analysis

The results of the multivariate logistic regression models mostly confirmed those of the univariate analysis. Indeed, PLP who reported consumption of at least 5 daily portions of fruit and vegetables (OR = 2.99, 95% CI = 1.13–7.86), that were minimally active (OR = 2.02, 95% CI = 1.01–4.07) and had at least one chronic disease (OR = 2.32, 95% CI = 1.18–4.55) were significantly more likely to have undergone FOBT(Model 1 in Table 2).

**Table 2:** Multiple logistic regression analysis investigating predictors of fecal occult blood test (FOBT) uptake in a screening program (Model 1) and willingness to undertake FOBT in prison (Model 2).

Model 1. FOBT uptake in a screening program°			
Log likelihood = −129.74, χ^2^ = 57.02 (16 df), *p* < 0.001, No. of obs = 279			
Variable	OR	95% CI	*p*
**Age group, years**			
≥61	1.00*		
56-60	0.31	0.12-0.74	0.009
50-55	0.47	0.23-0.95	0.036
**Institution**			
Prison 1	1.00*		
Prison 2	Backward elimination		
Prison 3	2.11	1.08-4.13	0.029
**Length of detention, years**			
>10	1.00*		
2-10	0.37	0.19-0.73	0.004
≤1	Backward elimination		
**At least one chronic disease**			
No	1.00*		
Yes	2.32	1.18-4.55	0.014
**Alcohol consumption (Audit-C)**			
Being at risk of alcohol abuse	1.00*		
Not being at risk of alcohol abuse	0.33	0.09-1.12	0.076
Never	0.27	0.08-0.88	0.030
**Physical activity (IPAQ score)**			
Active/very active	1.00*		
Minimally active	2.02	1.01-4.07	0.049
Inactive	1.51	0.59-3.85	0.386
**At least 5 daily portions of fruit and vegetables**			
No	1.00*		
Yes	2.99	1.13-7.86	0.027
**Two daily portions of dietary protein sources**			
No	1.00*		
Yes	0.49	0.26-0.94	0.031
**Sons/daughters**			
No	1.00*		
Yes	1.74	0.65-4.65	0.269
**Education level**			
None/primary school	0.67	0.29-1.52	0.334
Middle school	1.00*		
High school/university degree	Backward elimination		
**Body Mass Index (BMI) category**			
Obese	1.00*		
Overweight	0.52	0.27-1.01	0.052
Under/healthy weight	Backward elimination		
**Smoking status**			
Current smokers	1.00*		
Former smokers	Backward elimination		
Never smokers	1.74	0.74-4.09	0.205
**Rare consumption of snacks and sweets**			
No	1.00*		
Yes	1.53	0.79-2.95	0.199
**Model 2**. Willingness to uptake FOBT in prison^+^			
Log likelihood = -61.73, χ^2^ =64.45 (16 df), *p*<0.001, No. of obs =238			
**Variable**	**OR**	**95% CI**	***p***
**Institution**			
Prison 1	1.00*		
Prison 2	0.98	0.03-0.32	<0.001
Prison 3	Backward elimination		
**Length of detention, years**			
> 10	1.00*		
2-10	0.36	0.13-0.99	0.049
≤1	Backward elimination		
**Working activity in prison**			
No	1.00*		
Yes	4.18	1.18-14.84	0.027
**First detention**			
No	1.00*		
Yes	0.32	0.11-0.92	0.034
**Physical activity (IPAQ score)**			
Active/very active	1.00*		
Minimally active	0.27	0.08-0.89	0.031
Inactive	0.38	0.06-4.43	0.306
**Two daily portions of dietary protein sources**			
No	1.00*		
Yes	5.24	1.8-15.22	0.002
**FOBT uptake in a screening program**			
No	1.00*		
Yes	12.15	1.07-137.81	0.044
**Willingness to receive recommended vaccinations in prison**			
No	1.00*		
Yes	4.4	1.47-13.22	0.008
**Willingness to participate in interventions promoting healthy lifestyle in prison**			
No	1.00*		
Yes	0.28	0.09-0.79	0.016
**Age group, years**			
>60	1.00*		
56-60	0.57	0.2-1.6	0.288
50-55	Backward elimination		
**Education level**			
None/primary school	5.07	0.89-28.81	0.067
Middle school	1.00*		
High school/university degree	Backward elimination		
**BMI category**			
Obese	1.00*		
Overweight	0.29	0.06-1.42	0.129
Under/healthy weight	0.37	0.07-1.92	0.238
**Alcohol consumption (Audit-C)**			
Being at risk of alcohol abuse	1.00*		
Not being at risk of alcohol abuse	Backward elimination		
Never	2.54	0.95-6.81	0.064
**Rare consumption of snacks and sweets**			
No	1.00*		
Yes	0.36	0.13-1.02	0.055

* Reference category. ° The following variables were deleted by the backward elimination procedure: marital status, occupation before detention, working activity in prison, first detention. + The following variables were deleted by the backward elimination procedure: marital status, occupation before detention, sons/daughters, at least one chronic disease, smoking status, at least 5 daily portions of fruit and vegetables.

Moreover, those aged 50−55 years (OR = 0.47, 95% CI = 0.23–0.95) or 56−60 years (OR = 0.31, 95% CI = 0.12–0.74) compared to those aged > 60 years, who had a length of detention of 2−10 years (OR = 0.37, 95% CI = 0.19–0.73) compared to those with a longer duration (> 10 years), those who reported correct protein consumption (OR = 0.49, 95% CI = 0.26–0.94), and those who do not drink alcohol (OR = 0.27, 95% CI = 0.08–0.88) were significantly less likely to have ever undergone a FOBT for CRC screening purposes. Finally, significant differences in FOBT uptake were revealed in the investigated prisons (Model 1 in Table 2).

Willingness to participate in a FOBT screening program in prison was significantly higher in those involved in working activities in prison (OR = 4.18, 95% CI = 1.18–14.84), who had correct protein consumption (OR = 5.24, 95% CI = 1.8–15.22), who had expressed willingness to receive vaccinations in prison if offered (OR = 4.4, 95% CI = 1.47–13.22) and who had already undergone a FOBT in a screening program (OR = 12.15, 95% CI = 1.07–137.81) (Model 2 in Table 2). Moreover, those at their first experience of detention (OR = 0.32, 95% CI = 0.11–0.92), with a length of detention of 2−10 years (OR = 0.36, 95% CI = 0.13–0.99) compared to > 10 years, who were minimally active (OR = 0.27, 95% CI = 0.08–0.89), and had expressed their willingness to participate in interventions to promote healthy lifestyle (OR = 0.28, 95% CI = 0.09–0.79) were significantly less willing to undergo FOBT for screening purposes if offered in prison. Finally, significant differences about willingness were also detected in the different institutions (Model 2 in Table 2).

## Discussion

This is one of the few studies that has shed light on the CRC screening needs in PLP, as well as on their willingness to undergo the screening during their permanence in prison. The results have outlined a concerning but somehow expected scenario, since a very low ever attendance to CRC screening in the PLP has been found (25.2%), indicating a missed opportunity to prevent CRC in this population. However, it is promising that a consistent proportion of PLP (86%) expressed their willingness to undergo CRC screening if offered in prison. This finding stimulates the development of evidence-based interventions targeted to PLP for the achievement of the CRC screening threshold recommended by European Guidelines [34].

The result is even more concerning when considering the proportion of PLP who performed the CRC screening in the preceding two years (13.7%), given that the majority of PLP had an incarceration period that was longer than two years. However, it is interesting to note that among the ever attenders the percentage of those who underwent FOBT in prison (9.9%) was higher compared to those receiving it outside the prison in an organized program (8.6%) or for an opportunistic procedure (6.7%).

The very low rates of adherence to CRC screening in PLP are worrying, being lower than the values reported in the very few studies carried out in PLP, which found 31% [23] and 22.9% [25] PLP being up to date for CRC screening.

They are also lower compared to the Italian general population, with around 36% of Italians aged 50–74 years reporting to have performed CRC screening in the previous two years [5,35], although with very different regional adherence rates [35]. Indeed, in a recent study conducted in the same area on females in the general population, only one quarter of the eligible women reported to have ever undergone CRC screening [28].

The large proportion of non-attenders stimulates an in depth analysis of reported reasons for not having participated in CRC screening, which were in the majority related to lack of perception of risk and lack of having been advised by healthcare personnel. Indeed, lack of invitation to CRC screening pertains also to the general population, specifically in southern Italy, where invitation to CRC screening has increased from 43.5% in 2019 to 62% in 2022 [4], whereas adherence to invitation has decreased from 25.7% in 2019 to 19.5% in 2022 [4]. Moreover, it is alarming that only 12.8% reported to have been invited to participate in CRC screening during incarceration, given that PLP, according to the Italian legislation, should receive the same prevention and treatment interventions of all citizens, and are secured under the state responsibility.

However, it is promising that all of those who had been invited reported to have participated in the CRC screening, and that willingness to participate in CRC screening programs in prison was widely spread among PLP. These results confirm the positive attitude of PLP towards preventive programs to be provided in prison, which has already been reported for CRC screening in a qualitative study in England [26] and for other preventive activities, such as cervical cancer screening [36] and COVID-19 vaccination [37,38] in PLP in the same area.

It is also worthy of note to elucidate reasons reported by those who were not willing to participate, because most of them demonstrated definitely no interest in the test or considered it to be useless, suggesting that efforts are needed to improve PLP’s health literacy on the role of cancer prevention through screening test interventions.

Exploring factors associated with attendance and willingness to attend CRC screening programs allows to define barriers and facilitators to participation and suggests directions for the development of actions for promoting CRC screening adherence. The results of this study have shown that older age, the presence of chronic diseases, longer permanence in prison and correct consumption of fruit and vegetables were predictors of adherence to FOBT for screening purposes. Some of these findings have already been reported as determinants of CRC screening in the general population, such as a healthy lifestyle and presence of chronic disease, which may be a proxy for contact with the healthcare system, and therefore of a higher likelihood to receive a recommendation for screening [39]. In the same systematic review, the higher adherence of older subjects was suggested to be a reflection of the recognition of their high risk of getting and dying from CRC [39]. Contact with healthcare systems and older age were also among the most common factors identified in a systematic review of US studies investigating barriers and facilitators of CRC screening [40]. However, to the best of our knowledge no studies have investigated determinants of CRC screening in PLP, and the findings of this study provided evidence that duration of detention was a significant predictor of CRC screening, suggesting that permanence in prison may represent an opportunity to undergo CRC screening, although not yet consistently implemented.

Determinants of willingness to undergo CRC screening have shown that there seem to be a pattern of preventive activities that predicts the intention to participate in CRC screening, since having already participated in CRC screening, having the intention to undergo recommended vaccinations, being physically active and having a correct intake of proteins in the diet were all found to be determinants of willingness to participate in CRC screening. These results suggest the need to promote healthy behaviors in prisons, including correct nutrition and exercise, which is usually restricted by institutional conditions, such as limited access to outdoor areas and constrained schedules. Moreover, the findings that those who had more than one detention experience and with longer duration of detention were more willing to participate in CRC screening may be indirect evidence of the role that health services provided to PLP may have in the education to preventive activities.

Overall, the findings of this study have clearly demonstrated that, although there seem to be no substantial barriers to participation when it is proposed to PLP, CRC screening is still a missed opportunity in this disadvantaged population. These results are concerning, since it has been reported that, compared to those who are not incarcerated, PLP generally have later stage diagnoses for several cancer types, and this disparity is especially evident in screenable cancers, such as CRC, suggesting it could be driven by lower screening rates and thus less early detection [41]. Since in Italy, at the time of incarceration, PLP are provided medical examinations and all information regarding their health status are collected in the medical file, this could be an invaluable opportunity for the assessment of CRC screening needs, as well as all other primary and secondary prevention needs.

## Limitations

The results of this survey should be interpreted by acknowledging some potential limitations. First of all, the design was cross-sectional and hinders the inference on the cause-effect relationship between determinants and outcomes. In addition, data on CRC screening were self-reported, with no objective evaluation through clinical record and may have overestimated the real participation due to desirability bias. Furthermore, although probabilistic sampling methods have been applied, PLP voluntarily participated in the study, and since data on those who refused to participate were not available, it cannot be excluded that those who were more interested to preventive activities were more likely to participate in this survey. However, since response rate was very high, it is unlikely that the results have been affected by non-participation bias. Moreover, to reduce length of the interview, we had to make choices on information to be gathered, and even important data, such as family history of CRC, were not included in the questionnaire. It would be valuable to explore the impact of this variable in future studies. Finally, the participants were selected from three prisons in southern Italy and, therefore, caution should be taken for the generalizability of the findings to the whole population of PLP in Italy. Despite these limitations, this survey has provided interesting new knowledge on a neglected topic that may have substantial impact on PLP health.

In conclusion, the results of the study have highlighted that CRC screening adherence is very low in eligible PLP, and considering the positive attitude shown by PLP, there is substantial potential for improvements. Efforts should be directed to promoting PLP’s health literacy on the role of cancer prevention through screening test interventions, to the routine assessment of CRC screening needs in eligible PLP, and to the elimination of organizational barriers, which are peculiar to all healthcare activities provided to PLP.
